# Pharmacokinetics of sepantronium bromide (YM155), a small-molecule suppressor of survivin, in Japanese patients with advanced solid tumors: dose proportionality and influence of renal impairment

**DOI:** 10.1007/s00280-012-1913-z

**Published:** 2012-07-18

**Authors:** Yumiko Aoyama, Tetsuya Nishimura, Taiji Sawamoto, Taroh Satoh, Masataka Katashima, Kazuhiko Nakagawa

**Affiliations:** 1 Clinical Pharmacology, Development, Astellas Pharma Inc., 3-17-1, Hasune, Itabashi-ku, Tokyo, 174-8612 Japan; 2 Department of Frontier Science for Cancer and Chemotherapy, Graduate School of Medicine, Osaka University, Osaka, Japan; 3 Department of Medical Oncology, Kinki University, Faculty of Medicine, Osaka, Japan

**Keywords:** Sepantronium bromide, YM155, Pharmacokinetics, Renal impairment, Advanced solid tumor

## Abstract

**Purpose:**

The purpose of this analysis was to investigate the pharmacokinetics (PK) of sepantronium (YM155), a small-molecule suppressor of the expression of the antiapoptosis protein survivin, in Japanese patients with advanced solid tumors and to evaluate the effect of renal impairment on the PK profile of sepantronium.

**Methods:**

Sepantronium was administered as a continuous intravenous infusion of 1.8–10.6 mg/m^2^/day for 168 h (7 days) to 33 patients. PK parameters were estimated via non-compartmental method. Renal function was categorized for the analysis based on the chronic kidney disease guidance using eGFR values at pre-dose.

**Results:**

The PK of sepantronium was dose proportional in the dose range of 1.8–10.6 mg/m^2^/day. Age and sex did not significantly affect the PK of sepantronium. Results suggested that total clearance and renal clearance in patients with moderate renal impairment were 0.7-fold lower than those in patients with normal renal function, resulting in 1.3-fold higher steady-state concentration and area under the curve values. The PK parameters of sepantronium in patients with mild renal impairment were comparable to those in the patients with normal renal function.

**Conclusions:**

While age and sex did not significantly affect the PK of sepantronium, moderate renal impairment increased exposure of sepantronium by about 30 %. The results suggest that no dose adjustment is required for patients with mild renal impairment.

## Introduction

Sepantronium bromide (sepantronium, YM155, 1-(2-methoxyethyl)-2-methyl-4,9-dioxo-3-(pyrazin-2-ylmethyl)-4,9-dihydro-1*H*-naphtho[2,3-*d*]imidazolium bromide), a small-molecule survivin suppressant, was identified by cell-based, high-throughput screening and lead optimization. Sepantronium bromide selectively suppresses survivin expression, resulting in activation of caspases and apoptosis induction in hormone refractory prostate cancer cells. Sepantronium bromide showed broad spectrum antitumor activity and induced tumor regressions in various xenograft models rather than uncertainness of mode of action. Continuous infusion of sepantronium bromide has also been found to induce tumor regression and intratumoral survivin suppression in established human hormone refractory prostate cancer (HRPC), non-Hodgkin lymphoma (NHL), and non-small cell lung cancer (NSCLC) tumor xenografts [1–4].

Phase 2 studies to evaluate the safety, efficacy, and pharmacokinetics (PK) of sepantronium were conducted with a continuous intravenous infusion (CIVI) for 7 days and showed modest single-agent clinical activity in patients with NSCLC, HRPC, or unresectable stage III or IV melanoma, respectively [5–7]. Sepantronium is currently being investigated in phase 1 and phase 2 studies in combination therapy, enrolling patients with diffuse large B-cell lymphoma and other solid tumors [8–10].

The results of a monotherapy phase 1 study to evaluate tolerability, safety, efficacy, and PK of sepantronium in Japanese patients with advanced solid tumors have been reported previously [11]. Sepantronium was administered by CIVI for 7 days (168 h) every 21 days at 1.8, 3.6, 4.8, 6.0, 8.0, and 10.6 mg/m^2^/day. Sepantronium was generally well tolerated, and the maximum tolerated dose was estimated to be 8.0 mg/m^2^/day when administered as the CIVI for 7 days in Japanese patients. Dose-limiting toxicities (DLT) observed in the study were increased blood creatinine, grade 3 increased serum aspartate aminotransferase (AST), and grade 4 anemia. Steady-state conditions were achieved 24 h after start of infusion for all doses administered. The concentrations of unchanged drug declined rapidly following a biphasic manner after termination of infusion. It appeared that systemic exposure increased with increasing doses and no accumulation was noted with repeated doses. Mean values for elimination half-life (*t*_1/2_) and total body clearance (CL) of sepantronium seemed constant across the dose ranges. The urinary excretion ratio of unchanged drug ranged from 25 to 42 % and showed no relationship with the dose administered. Non-clinical studies in rats showed that three metabolites were identified in bile and urine after a single intravenous dose of sepantronium; however, sepantronium was minimally metabolized when incubated with human cryopreserved hepatocytes [12].

Given these characteristics, it is anticipated that a substantial reduction in renal function may affect the renal clearance of sepantronium, and consequently its PK, as well as possibly, its safety, and tolerability.

The present analysis was performed to evaluate the effect of renal impairment on PK of sepantronium using a linear mixed effect model with data obtained from a previously reported phase 1 study in Japan. In addition, dose proportionality of sepantronium was evaluated, and an exploratory analysis to investigate the effect of demographics on the PK of sepantronium was performed.

## Methods

A retrospective analysis of data obtained from the previously reported phase 1 study in Japanese subjects [11] was performed.

### Study design

The study was an open-label, single center, phase 1, dose-escalation study with administration of CIVI dose of sepantronium as monotherapy over 7 days (168 h) every 21 days. The safety, tolerability, efficacy, and PK of sepantronium were evaluated in male and female Japanese patients with advanced solid tumors. Sepantronium was prepared for administration by dilution of the appropriate volume of concentrated stock solution in 5 % dextrose in a light- and temperature-controlled environment.

The study consisted of 6 dose cohorts of 3–6 patients each treated with 1.8, 3.6, 4.8, 6.0, 8.0, or 10.6 mg/m^2^/day. Doses were expressed as those of the cationic moiety of sepantronium bromide. Each 21-day cycle included a 7-day (168-h) administration period and a 14-day observation period (1 cycle). This study was conducted at the Department of Medical Oncology, Kinki University Hospital, Osaka, Japan. The protocol was approved by an independent ethics committee for the study site, and the study was conducted in accordance with the principles of the Declaration of Helsinki. Results regarding the tolerability, safety, and basic PK profile of sepantronium have been reported previously [11].

### Population

The study enrolled Japanese male and female patients with advanced solid tumors. Eligibility criteria for patients enrolled in the study included refractory advanced solid tumors for which no standard therapy was available; histologic or cytologic diagnosis of cancer; age at least 20 years; life expectancy of at least 12 weeks; Eastern Cooperative Oncology Group performance status of <3; and adequate hematopoietic, hepatic, and renal functions (absolute neutrophil count of ≥1.5 × 10^9^/L, platelets of ≥100 × 10^9^/L, hemoglobin of ≥9 g/dL, bilirubin within 1.5 × upper limit of normal, transaminases of ≤2.5 × upper limit of normal, and creatinine of ≤ upper limit of normal) [11].

### Blood and urine sampling

Venous blood samples were collected in tubes containing heparin sodium from a site other than the infusion site before and at 0.25, 0.5, 1, 2, 3, 4, 6, 12, 24, 48, 72, 96, 120, and 144 h after start of infusion, as well as at the end of infusion (168 h), and at the following time points thereafter: 168.25, 168.5, 169, 170, 171, 172, 174, 180, 192, and 216 h after the start of infusion. Blood samples were centrifuged immediately, and the plasma samples obtained were stored at −20 °C before analysis. To determine the urinary concentration of unchanged sepantronium, urine samples were collected over the 216-h period after start of CIVI and stored at −20 °C before analysis. Blood and urine samples for PK evaluation were collected during cycle 1 and cycle 2 [11].

### Bioanalytical procedures

Measurement of sepantronium concentration in plasma and urine samples was performed by Astellas Europe B.V. EDD using liquid chromatography tandem mass spectrometry (LC–MS/MS). The lower limit of quantitation for sepantronium was 0.05 ng/mL in plasma and 1.0 ng/mL in urine. Concentrations were expressed as those of the cationic moiety of sepantronium bromide [11, 13]. The precision and the accuracy of inter- and intra-assay for the LC–MS/MS methods were within ±20 % (unpublished data).

### Pharmacokinetic analysis

PK parameters of sepantronium in plasma and urine were calculated using WinNonlin Professional^®^ version 5.0.1 (Pharsight Corporation, Mountain View, CA, USA) and the SAS^®^ system (SAS Institute Inc., Cary, NC, USA). The area under the curve (AUC) was calculated according to the linear trapezoidal rule from zero to time t of the last measurable concentration above the lower limit of quantitation. Steady-state concentration (C_SS_) was the mean value of daily concentrations taken through 7-day CIVI (the mean value of concentration at 24, 48, 72, 96, 120, 144, and 168 h after start of infusion). Terminal elimination half-life (*t*_1/2_) of sepantronium was calculated as follows: *t*_1/2_ = ln 2/terminal elimination rate constant. CL is the total systemic clearance, estimated by: CL = total amount of dose/AUC from time zero to infinity. CL_*R*_ is renal clearance, estimated by: CL_*R*_ = cumulative amount excreted in urine/AUC. *V*_*d*_ is apparent volume of distribution, estimated by: *V*_*d*_ = CL/terminal elimination rate constant. Ae is amount excreted in urine. Fe is fraction excreted in urine estimated by: Fe = Ae/Dose.

### Statistical analysis

Statistical analysis was performed using the SAS^®^ system. Dose proportionality in PK parameters of sepantronium was evaluated via power model regression using a mixed effect model. The effect of cycle, dose, and demographics (age and sex) upon PK parameters as a fixed effect was investigated.

Renal function was categorized into normal renal function with eGFR of ≥90 mL/min/1.73 m^2^ (normal group), mild decrease in eGFR (60–89 mL/min/1.73 m^2^) (mild renal impairment group), or moderate decrease in eGFR (30–59 mL/min/1.73 m^2^) (moderate renal impairment group) based on the chronic kidney disease guidance [14]. The eGFR for a Japanese population was calculated using the following equation [15]:

$$ {\text{eGFR}}\left( {{\text{mL}}/{ \min }/ 1. 7 3 \, {\text{m}}^{ 2} } \right) = 1 9 4\times \left( {\text{serum creatinine}} \right)^{ - 1.0 9 4} \times \left( {\text{age}} \right)^{ - 0. 2 8 7} \left( { \times 0. 7 3 9 {\text{ if female}}} \right) $$

PK parameters among renal function groups were compared using geometric mean ratios (GMRs) and their 90 % confidence intervals (CIs) with renal function and cycle as fixed effects and subject as a random effect in the mixed effect model. Results of patients with normal eGFR (normal group) were considered as reference data. Analysis was performed using natural-log transformed PK parameters. The point estimates and their 90 % CIs were exponentiated, and the results were presented as a natural scale. Analysis was performed using dose-normalized AUC (AUC/Dose), dose-normalized C_SS_ (C_SS_/Dose), CL, and CL_*R*_.

## Results

### Study populations

Patient characteristics are summarized in Table 1. A total of 33 male (*n* = 23) and female (*n* = 10) patients with advanced solid tumors received sepantronium as a CIVI for at least 1 cycle. The PK was evaluated based on data from cycle 1 and cycle 2. After exclusion of 1 patient who had no concentration data, 32 patients were included in the analysis.

**Table 1:** Patient characteristics

Descriptive statistics for patient demographics		Number of PK data sets and frequency of renal function			
	No. of patients		Cycle 1	Cycle 2	Total
Total patients	33	Total	31	15	46
Male/female	23/10	Renal function			
Age (years)		Normal	12	7	19
Median (range)	59 (26–81)	Mild	14	7	21
Body weight (kg)		Moderate	5	1	6
Median (range)	54 (40–88)				
BSA					
Median (range)	1.6 (1.3–2.0)				

Thirty-two of 33 patients who received sepantronium administration had concentration data and were PK evaluable patients. Fourteen of 32 had both cycle 1 and cycle 2 data, 17 patients had cycle 1 data, and remaining 1 patient had cycle 2 data

Fourteen of 32 patients had both cycle 1 and cycle 2 data, while the 17 patients had cycle 1 data and remaining 1 had cycle 2 data, yielding a total of 46 data sets for use in the analysis. The baseline of serum creatinine for enrolled patients ranged from 0.4 mg/dL to 1.2 mg/dL, and were under the upper limit of normal level (1.3 mg/dL). The number of data sets in moderate renal impairment group (30–59 mL/min/1.73 m^2^) was 6. The number of data sets in normal group (≥90 mL/min/1.73 m^2^) (*n* = 19) and mild renal impairment group (60–89 mL/min/1.73 m^2^) (*n* = 21) was comparable. All dose cohorts included normal group and mild renal impairment group. Moderate renal impairment group were in the dose cohorts of 3.6 (*n* = 3), 4.8 (*n* = 1), or 6.0 mg/m^2^/day (*n* = 2). There were no patients who had severe decrease in eGFR or who required dialysis. Renal function in 2 patients changed from cycle 1 to cycle 2 (from mild to normal in one patient and vice versa in the other).

### Pharmacokinetics

**Fig. 1:**
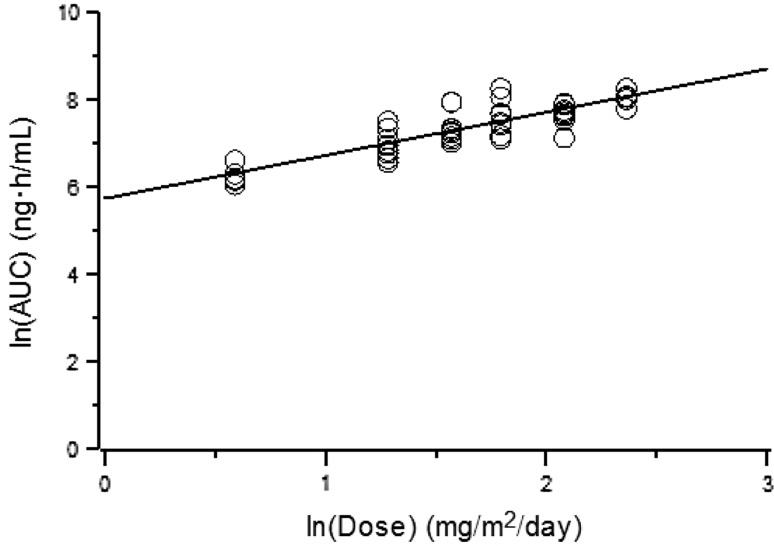
Relationship between dose and AUC of sepantronium. *Line*: power model regression, *empty circle*: individual value. Slopes and their 90 % confidence intervals obtained from power model regression between the dose and AUC at a dose range of 1.8–10.6 mg/m^2^/day was 0.981 (0.868–1.094)

Descriptive statistics for PK parameters of sepantronium by dose cohort were presented in Table 2. The relationship between dose and AUC was presented in Fig. 1.

**Table 2:** Descriptive Statistics for PK Parameters

	Cohort 1 1.8 mg/m^2^/day (*n* = 6)	Cohort 2 3.6 mg/m^2^/day (*n* = 10)	Cohort 3 4.8 mg/m^2^/day (*n* = 9)	Cohort 4 6.0 mg/m^2^/day (*n* = 8)	Cohort 5 8.0 mg/m^2^/day (*n* = 8)	Cohort 6 10.6 mg/m^2^/day (*n* = 5)
AUC (ng h/mL)	538 ± 119	1,186 ± 385	1,738 ± 685	2,239 ± 952	2,233 ± 489	3,235 ± 526
*t*_1/2_ (h)	7 ± 3	21 ± 9	15 ± 10	16 ± 6	21 ± 9	29 ± 14
CL (L/h)	42 ± 7	39 ± 13	35 ± 11	34 ± 11	41 ± 14	34 ± 7
C_SS_ (ng/mL)	3 ± 1	7 ± 2	10 ± 4	14 ± 6	13 ± 3	19 ± 3
*V*_*d*_ (L)	436 ± 175	1,197 ± 568	759 ± 541	795 ± 347	1,169 ± 484	1,544 ± 955

	(*n* = 6)	(*n* = 6)	(*n* = 9)	(*n* = 7)	(*n* = 4)	(*n* = 4)
Ae (mg)	7 ± 1	16 ± 5	16 ± 5	19 ± 6	25 ± 2	46 ± 8
Fe (%)	31 ± 4	35 ± 8	30 ± 9	28 ± 6	29 ± 4	42 ± 8

Values are mean ± standard deviation. *AUC*, area under the curve from zero to time t of the last measurable concentration above the limit of quantitation: *t*_1/2_, terminal elimination half-life, *CL* total systemic clearance, *C*_*SS*_ steady-state concentration, *V*_*d*_ apparent volume of distribution, *Ae* amount excreted in urine, *Fe* fraction excreted in urine

There was no difference in PK parameters between cycle 1 and cycle 2 [11], and the analysis was performed using combined data from cycle 1 and cycle 2. Inter-individual variability of sepantronium PK was moderate as shown in Table 2. Slopes [90 % CIs] by power model regression for AUC and C_SS_ versus dose were 0.981 [0.868–1.094] and 0.998 [0.885–1.110], respectively. The results suggested that AUC and C_SS_ increased in a dose proportional manner.

Significance as a fixed effect of age and sex was evaluated by adding to the power model; however, for these models, either fit statistics were not improved by adding age and sex as fixed effects, or the effect was not significant (age, *p* > 0.1; sex, *p* > 0.04). No demographics were therefore added to the model as a fixed effect.

**Fig. 2:**
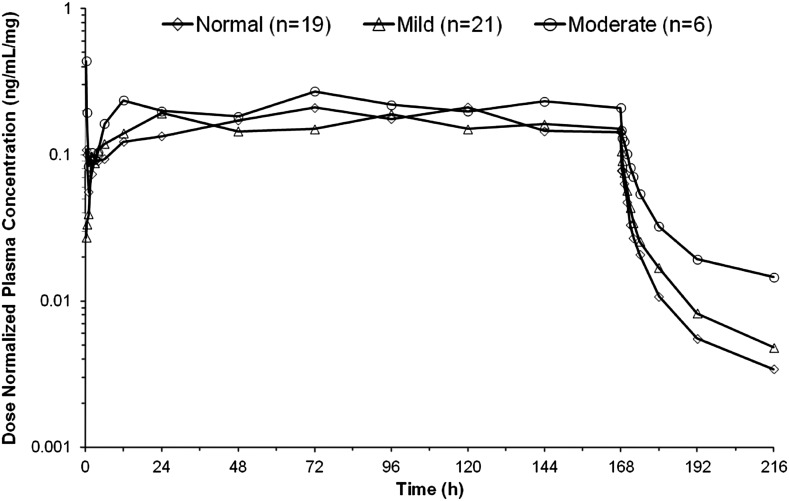
Mean dose-normalized plasma concentration versus time profile of sepantronium after continuous intravenous infusion of 1.8–10.6 mg/m^2^/day for 168 h in Japanese patients with advanced solid tumors

**Fig. 3:**
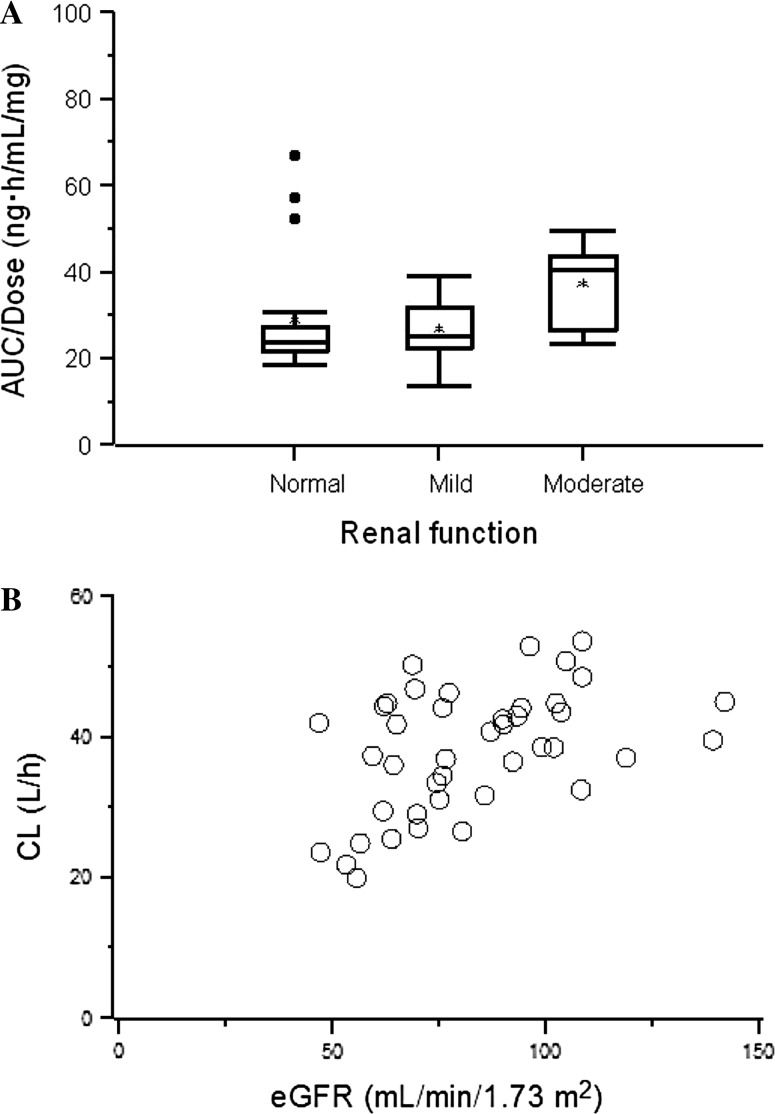
Relationship between renal function and PK parameters of sepantronium. *Star* represents a mean value: *box* represents a range of 50 % interval: a *bar* in each *box* represents a median value: *bar* under the *box* represents a 25 percentile: *bar* over the *box* represents a 75 percentile: *fixed circle* represents outlier in **a**. Pearson’s correlation coefficient between CL and eGFR = 0.46, *p* = 0.0021 in **b**

Summary statistics of PK parameters by renal function are presented in Table 3. Mean plasma concentration versus time profile of sepantronium is presented in Fig. 2. The relationship between PK parameter and renal function is presented in Fig. 3.

**Table 3:** Summary of pharmacokinetic parameters of sepantronium after continuous intravenous infusion of 1.8, 3.6, 4.8, 6.0, 8.0, or 10.6 mg/m^2^/day for 168 h

PK parameters (mean ± SD)	Normal (*n* = 19)	Mild (*n* = 21)	Moderate (*n* = 6)
AUC/Dose (ng h/mL/mg)	29 ± 14	27 ± 7	37 ± 10
C_SS_/Dose (ng/mL/mg)	0.17 ± 0.08	0.16 ± 0.04	0.22 ± 0.07
CL (L/h)	39 ± 11	39 ± 11	28 ± 9

	(*n* = 14)	(*n* = 14)	(*n* = 4)
CL_*R*_ (L/h)	13 ± 5	13 ± 2	9 ± 4

PK Parameter comparison, GMR (90 % CI)	Mild/normal	Moderate/normal
AUC/Dose (ng h/mL/mg)	0.976 (0.819–1.162)	1.340 (1.033–1.738)
C_SS_/Dose (ng/mL/mg)	0.989 (0.824–1.187)	1.273 (0.969–1.672)
CL (L/h)	1.021 (0.857–1.217)	0.740 (0.570–0.960)
CL_*R*_ (L/h)	1.107 (0.887–1.383)	0.695 (0.499–0.968)

*SD* standard deviation, *AUC/Dose* dose-normalized area under the curve from zero to time t of the last measurable concentration above the limit of quantitation, *C*_*SS*_ */Dose* dose-normalized steady-state concentration, *CL* total systemic clearance, *CL*_*R*_ renal clearance, *GMR* geometric mean ratio, *CI* confidence interval

Mean plasma concentrations in the moderate renal impairment group were slightly higher than the concentrations in other groups after termination of the sepantronium infusion. Mean PK parameters in the mild impairment group were comparable to those in the normal group. The GMR for the mild renal impairment group versus normal group was nearly equal to 1, and the 90 % CIs were almost in the range of 0.8–1.25. Renal function in 2 patients changed from cycle 1 to cycle 2 (from mild to normal in one and vice versa in the other), and PK parameters in these patients were similar for both cycles. Results for the moderate renal impairment group showed lower mean CL and CL_*R*_ compared to the normal group, and AUC/Dose and C_SS_/Dose in the moderate renal impairment group were 1.3-fold higher than those in the normal group.

Two of the five patients in the highest dose cohort (10.6 mg/m^2^/day) had DLT of increased blood creatinine. Of note is the fact that these two patients with the DLT had mild renal impairment. The AUC values of these two patients were 3975 and 3098 ng h/mL, respectively, and were equal to or greater than the values in the other patients in the same dose cohort (2510–3351 ng h/mL).

## Discussion

We evaluated the effect of renal impairment on PK of sepantronium in patients with advanced solid tumors using the data obtained from an open-label, phase 1 study. Sepantronium was administered as CIVI at a dose and rate of 1.8–10.6 mg/m^2^/day over 7 days. Overall, PK parameters of sepantronium were similar in patients with mild impairment and patients with normal renal function; however, patients with moderate impairment had a slightly lower clearance of sepantronium. The GMR for the mild renal impairment group compared to the normal group was nearly equal to 1, and the 90 % CIs were in the range of 0.8–1.25 which is commonly accepted as an equivalence range. The results indicated that there was no clinically significant difference in PK between patients with normal renal function and patients with mild renal impairment.

Two phase 1 studies have consistently reported excretion ratios of sepantronium as unchanged drug into urine of approximately 30 %, results which indicate that urinary excretion is an important elimination routes of sepantronium [11, 13]. The results that moderate renal impairment reduced the CL_*R*_ of sepantronium and mild renal impairment had no effect on the PK are in agreement with the above assumption.

Impaired renal function often alters a drug’s PK profile, when the drug is eliminated primarily by renal excretion. In vitro and animal studies have suggested that renal impairment may affect or down-regulate various CYP enzymes and transporters that may lead to clinically relevant changes in non-renal clearance [16, 17]. The CL and CL_*R*_ of sepantronium decreased in parallel in the moderate renal impairment group in the present analysis. Although the elimination mechanism of sepantronium is yet not fully known, it is assumed that a decrease in CL reflects a reduction in CL_*R*_ linearly.

The majority of the PK data sets were comprised of normal group (*n* = 19) and mild renal impairment group (*n* = 21). Only 6 were in the moderate renal impairment group. None of the patients had severe renal impairment. Inter-subject variability of sepantronium PK parameters was moderate as shown in Table 3, and there were three outliers in the normal renal function group which were presented as fixed circle in Fig. 3a. Figure 3b shows a relationship between the eGFR and CL of sepantronium. A weak correlation was observed between eGFR and CL of sepantronium when outliers were excluded (Pearson’s correlation coefficient between CL and eGFR = 0.46, *p* = 0.0021). There is a possibility that a strong relationship will be observed between eGFR and CL of sepantronium if further investigation is conducted using comparable number of patients with moderate and severe renal impairment compared to patients with normal renal function.

Most patients enrolled in the study had normal serum alanine aminotransferase and AST values, and an analysis to evaluate the effect of hepatic impairment on PK of sepantronium was not performed. Of interest was a weak correlation observed between the CL_*R*_ and baseline value of alkaline phosphatase (ALP), which was above upper normal range in 15 of 46 PK data sets at the baseline (Pearson’s correlation coefficient = 0.40, *p* = 0.0229). However, no similar relationship was observed between CL and ALP (Pearson’s correlation coefficient = 0.15, *p* = 0.3106). The reason for this finding is unclear.

It was reported that 3 metabolites were identified in bile and urine samples obtained after a single intravenous dose of sepantronium to rats. The proposed metabolic pathways of sepantronium in rats involve *N*-dealkylation, *o*-demethylation, and the oxidation of a methyl group to a carboxylic acid. Sepantronium is minimally metabolized when incubated with human cryopreserved hepatocyte [12]. It was suggested that human organic cation transporter 1 (OCT1) was the predominant transporter for the hepatic uptake of sepantronium, and that excretion into bile was an important elimination pathway of sepantronium in humans. It has also been reported that the transporter-mediated uptake clearance observed in vitro may account for the in vivo intrinsic hepatic clearance [18]. The contribution ratio of hepatic metabolism to metabolic clearance in humans is unclear presently; however, given these results from in vitro and non-clinical studies, the contribution ratio of hepatic metabolism in humans is likely small. Further investigation for metabolites in human will help to clarify the effect of hepatic impairment on PK of sepantronium.

Frequently, the effects of renal impairment are clarified by conducting a clinical study enrolling patients with renal impairment and comparing them with those in matched healthy subjects, or by performing a model analysis using a non-linear mixed effect model [17]. The present analysis is another approach to investigate the effect of renal or hepatic impairment on the PK of compounds early in the clinical trials.

Dose proportionality of the PK of sepantronium was evaluated via the power model regression. Slopes and their 90 % CIs for AUC and C_SS_ versus dose were within the range of 0.8–1.25, indicating that exposure of sepantronium increased in a linear dose proportional manner. Other PK parameters were similar among dose cohorts. These results suggested that the PK of sepantronium was linear at a dose range from 1.8 to 10.6 mg/m^2^/day. In agreement with the present findings, an earlier phase 1 study for sepantronium conducted in the United States found that the values of C_SS_ and AUC increased in a dose proportional manner, and CL was independent of dose over the range of 1.8 to 4.8 mg/m^2^/day [13]. The effect of cycle on sepantronium PK was evaluated by adding cycle numbers to the model as a fixed effect; however, the effect was not significant (*p* > 0.1). These results indicate that there was no difference in PK parameters between cycle 1 and cycle 2 and accumulation with repeated dosing was not observed. Demographics such as age and sex did not significantly affect the PK of sepantronium.

A previous non-clinical toxicology study found that short-term exposure at high plasma concentrations caused nephrotoxicity [11]. Two of five patients in the highest dose cohort (10.6 mg/m^2^/day) had DLT of increased blood creatinine [11]. Of note is the fact that these two patients with the DLT had mild renal impairment. The AUC values of these 2 patients were equal or greater than the values in the other patients in the same dose cohort. The PK of sepantronium was linear, and individual AUC and C_SS_ values in lower-dose cohorts were not in excess of those patients receiving 10.6 mg/m^2^/day, although inter- and intra-patient variability was moderate.

In conclusion, while age and sex did not significantly affect the PK of sepantronium; moderate renal impairment increased exposure of sepantronium by about 30 %. The CL and CL_*R*_ of sepantronium were lower in patients with moderate renal impairment relative to the patients with normal renal function. The PK in patients with mild renal impairment was comparable to those for patients with normal renal function. The results suggest that no dose adjustment is required for patients with mild renal impairment. It will be necessary to monitor the safety in patients with moderate renal impairment.
